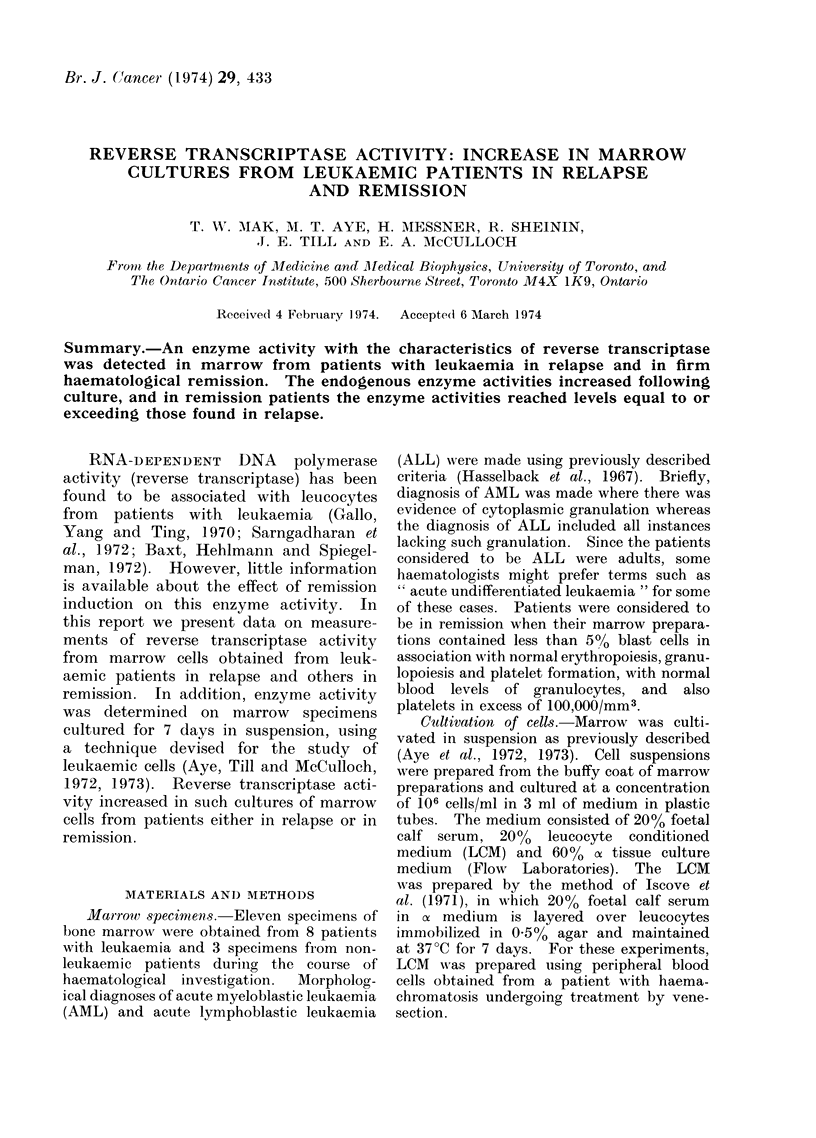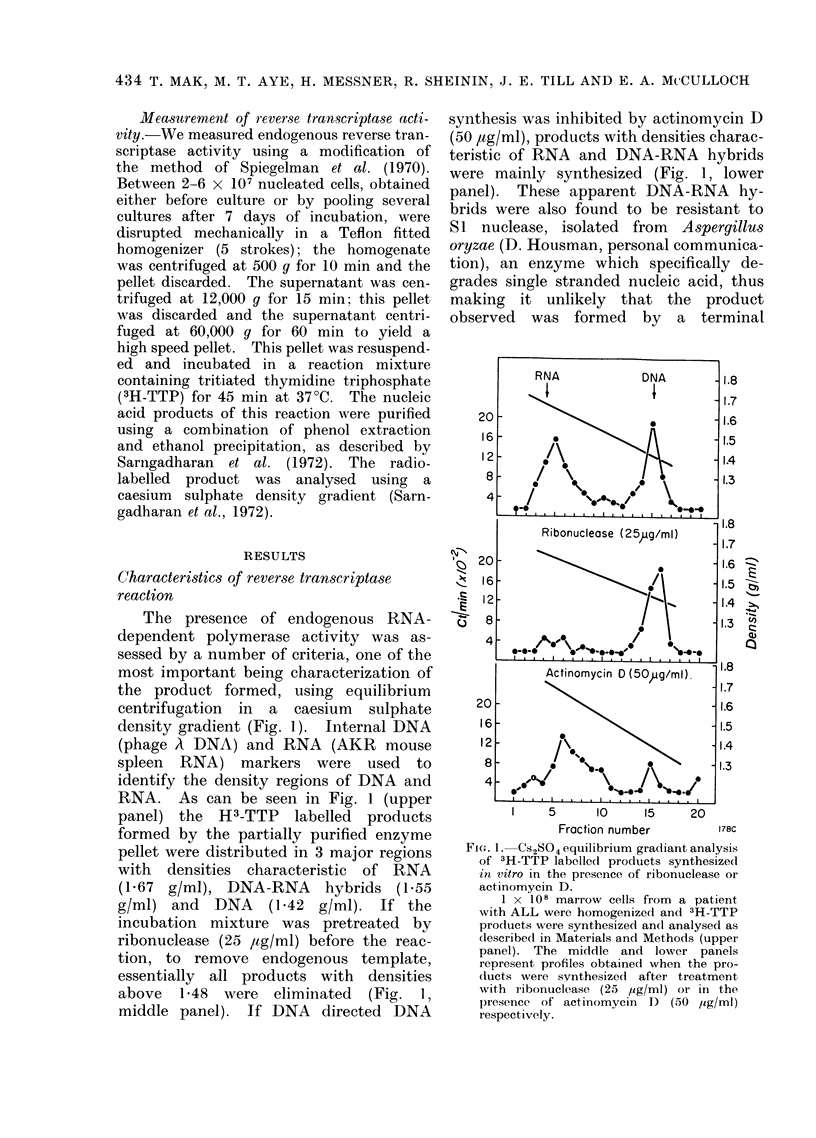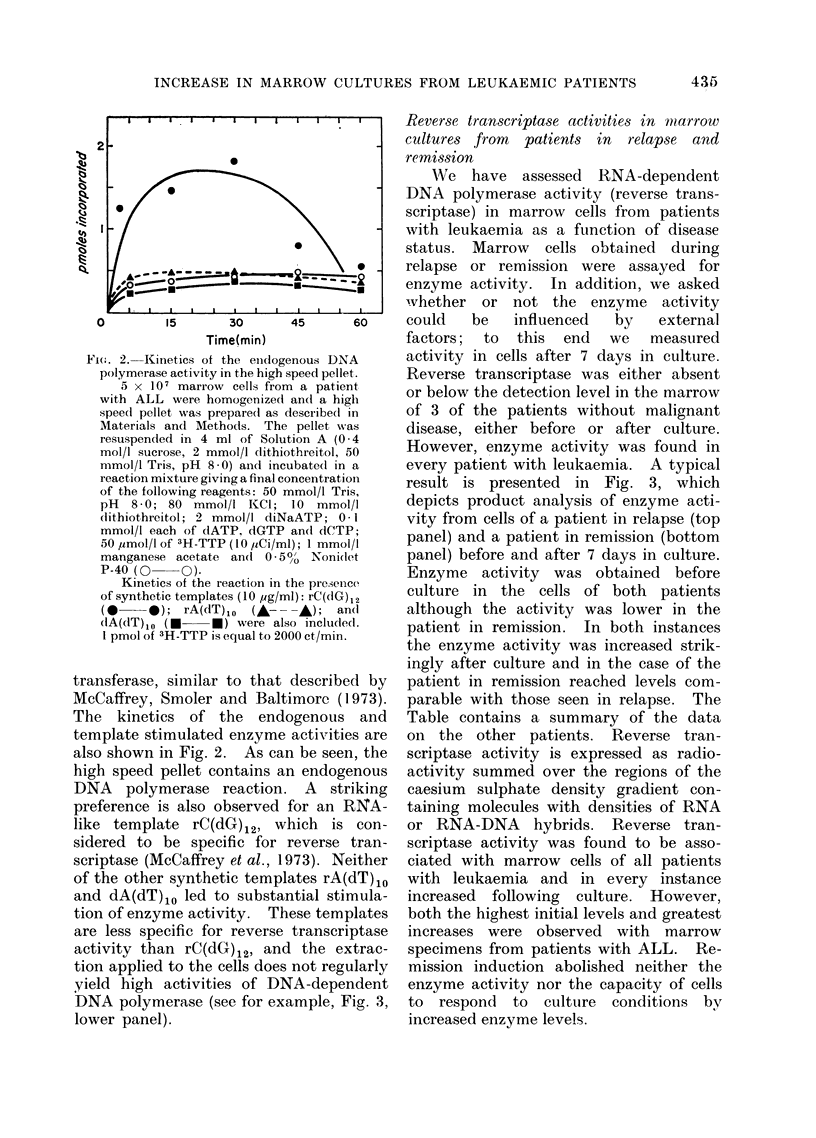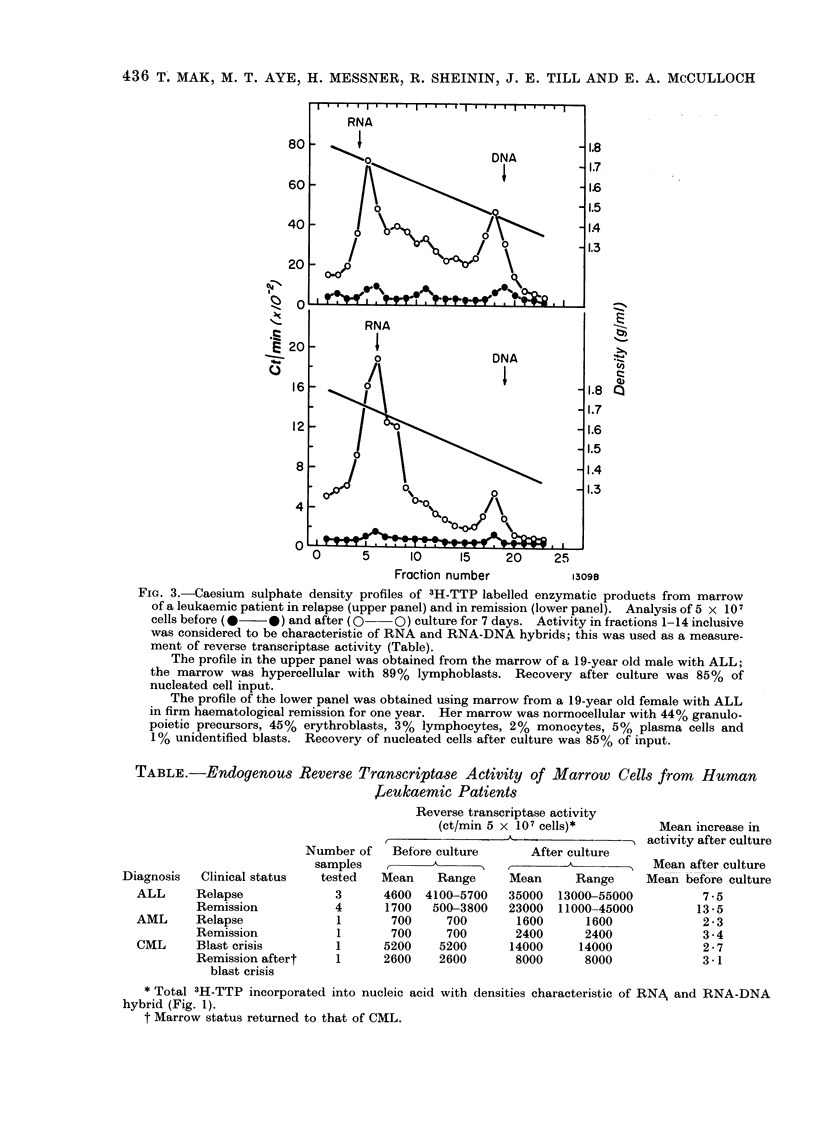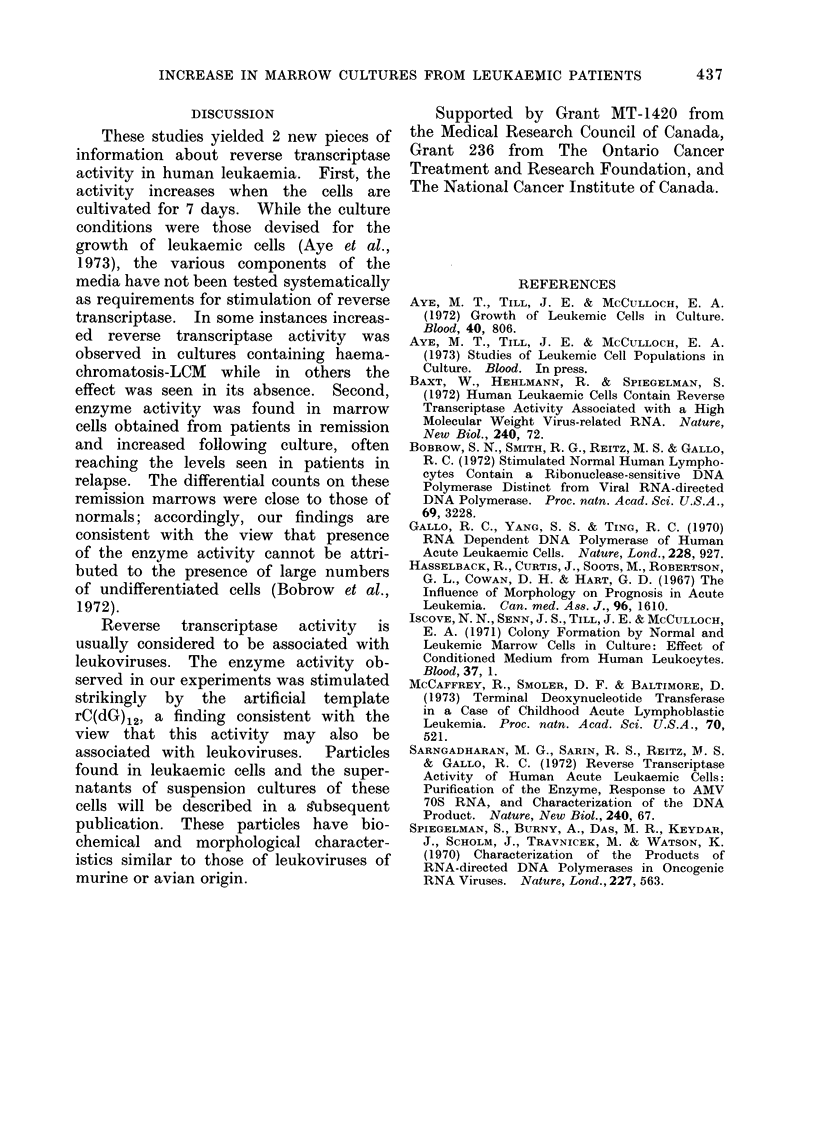# Reverse Transcriptase Activity: Increase in Marrow Cultures from Leukaemic Patients in Relapse and Remission

**DOI:** 10.1038/bjc.1974.95

**Published:** 1974-06

**Authors:** T. W. Mak, M. T. Aye, H. Messner, R. Sheinin, J. E. Till, E. A. McCulloch

## Abstract

An enzyme activity with the characteristics of reverse transcriptase was detected in marrow from patients with leukaemia in relapse and in firm haematological remission. The endogenous enzyme activities increased following culture, and in remission patients the enzyme activities reached levels equal to or exceeding those found in relapse.


Br. J. Cancer (1974) 29, 433

### REVERSE TRANSCRIPTASE ACTIVITY: INCREASE IN MARROW CULTURES FROM LEUKAEMIC PATIENTS IN RELAPSE AND REMISSION

T. W. MAK, M. T. AYE, H. MESSNER, R. SHEININ, J. E. TILL AND E. A. McCULLOCH

*From the Departments of Medicine and Medical Biophysics, University of Toronto, and The Ontario Cancer Institute, 500 Sherbourne Street, Toronto M4X 1K9, Ontario*

Received 4 February 1974. Accepted 6 March 1974

**Summary.—An enzyme activity with the characteristics of reverse transcriptase was detected in marrow from patients with leukaemia in relapse and in firm haematological remission. The endogenous enzyme activities increased following culture, and in remission patients the enzyme activities reached levels equal to or exceeding those found in relapse.**

RNA-DEPENDENT DNA polymerase activity (reverse transcriptase) has been found to be associated with leucocytes from patients with leukaemia (Gallo, Yang and Ting, 1970; Sarngadharan *et al.*, 1972; Baxt, Hehlmann and Spiegelman, 1972). However, little information is available about the effect of remission induction on this enzyme activity. In this report we present data on measurements of reverse transcriptase activity from marrow cells obtained from leukaemic patients in relapse and others in remission. In addition, enzyme activity was determined on marrow specimens cultured for 7 days in suspension, using a technique devised for the study of leukaemic cells (Aye, Till and McCulloch, 1972, 1973). Reverse transcriptase activity increased in such cultures of marrow cells from patients either in relapse or in remission.

### MATERIALS AND METHODS

*Marrow specimens.*—Eleven specimens of bone marrow were obtained from 8 patients with leukaemia and 3 specimens from non-leukaemic patients during the course of haematological investigation. Morphological diagnoses of acute myeloblastic leukaemia (AML) and acute lymphoblastic leukaemia (ALL) were made using previously described criteria (Hasselback *et al.*, 1967). Briefly, diagnosis of AML was made where there was evidence of cytoplasmic granulation whereas the diagnosis of ALL included all instances lacking such granulation. Since the patients considered to be ALL were adults, some haematologists might prefer terms such as " acute undifferentiated leukaemia " for some of these cases. Patients were considered to be in remission when their marrow preparations contained less than 5% blast cells in association with normal erythropoiesis, granulopoiesis and platelet formation, with normal blood levels of granulocytes, and also platelets in excess of $100{,}000/mm^3$.

*Cultivation of cells.*—Marrow was cultivated in suspension as previously described (Aye *et al.*, 1972, 1973). Cell suspensions were prepared from the buffy coat of marrow preparations and cultured at a concentration of $10^6$ cells/ml in 3 ml of medium in plastic tubes. The medium consisted of 20% foetal calf serum, 20% leucocyte conditioned medium (LCM) and 60% $\alpha$ tissue culture medium (Flow Laboratories). The LCM was prepared by the method of Iscove *et al.* (1971), in which 20% foetal calf serum in $\alpha$ medium is layered over leucocytes immobilized in 0·5% agar and maintained at 37°C for 7 days. For these experiments, LCM was prepared using peripheral blood cells obtained from a patient with haemachromatosis undergoing treatment by venesection.

*Measurement of reverse transcriptase activity.*—We measured endogenous reverse transcriptase activity using a modification of the method of Spiegelman *et al.* (1970). Between 2–6 × $10^7$ nucleated cells, obtained either before culture or by pooling several cultures after 7 days of incubation, were disrupted mechanically in a Teflon fitted homogenizer (5 strokes); the homogenate was centrifuged at 500 *g* for 10 min and the pellet discarded. The supernatant was centrifuged at 12,000 *g* for 15 min; this pellet was discarded and the supernatant centrifuged at 60,000 *g* for 60 min to yield a high speed pellet. This pellet was resuspended and incubated in a reaction mixture containing tritiated thymidine triphosphate ($^3$H-TTP) for 45 min at 37°C. The nucleic acid products of this reaction were purified using a combination of phenol extraction and ethanol precipitation, as described by Sarngadharan *et al.* (1972). The radiolabelled product was analysed using a caesium sulphate density gradient (Sarngadharan *et al.*, 1972).

### RESULTS

### *Characteristics of reverse transcriptase reaction*

The presence of endogenous RNA-dependent polymerase activity was assessed by a number of criteria, one of the most important being characterization of the product formed, using equilibrium centrifugation in a caesium sulphate density gradient (Fig. 1). Internal DNA (phage λ DNA) and RNA (AKR mouse spleen RNA) markers were used to identify the density regions of DNA and RNA. As can be seen in Fig. 1 (upper panel) the $H^3$-TTP labelled products formed by the partially purified enzyme pellet were distributed in 3 major regions with densities characteristic of RNA (1·67 g/ml), DNA-RNA hybrids (1·55 g/ml) and DNA (1·42 g/ml). If the incubation mixture was pretreated by ribonuclease (25 μg/ml) before the reaction, to remove endogenous template, essentially all products with densities above 1·48 were eliminated (Fig. 1, middle panel). If DNA directed DNA synthesis was inhibited by actinomycin D (50 μg/ml), products with densities characteristic of RNA and DNA-RNA hybrids were mainly synthesized (Fig. 1, lower panel). These apparent DNA-RNA hybrids were also found to be resistant to S1 nuclease, isolated from *Aspergillus oryzae* (D. Housman, personal communication), an enzyme which specifically degrades single stranded nucleic acid, thus making it unlikely that the product observed was formed by a terminal

FIG. 1.—$Cs_2SO_4$ equilibrium gradiant analysis of $^3$H-TTP labelled products synthesized *in vitro* in the presence of ribonuclease or actinomycin D.

1 × $10^8$ marrow cells from a patient with ALL were homogenized and $^3$H-TTP products were synthesized and analysed as described in Materials and Methods (upper panel). The middle and lower panels represent profiles obtained when the products were synthesized after treatment with ribonuclease (25 μg/ml) or in the presence of actinomycin D (50 μg/ml) respectively.

FIG. 2.—Kinetics of the endogenous DNA polymerase activity in the high speed pellet.

$5 \times 10^7$ marrow cells from a patient with ALL were homogenized and a high speed pellet was prepared as described in Materials and Methods. The pellet was resuspended in 4 ml of Solution A (0·4 mol/l sucrose, 2 mmol/l dithiothreitol, 50 mmol/l Tris, pH 8·0) and incubated in a reaction mixture giving a final concentration of the following reagents: 50 mmol/l Tris, pH 8·0; 80 mmol/l KCl; 10 mmol/l dithiothreitol; 2 mmol/l diNaATP; 0·1 mmol/l each of dATP, dGTP and dCTP; 50 μmol/l of $^3$H-TTP (10 μCi/ml); 1 mmol/l manganese acetate and 0·5% Nonidet P-40 (○——○).

Kinetics of the reaction in the presence of synthetic templates (10 μg/ml): $rC(dG)_{12}$ (●——●); $rA(dT)_{10}$ (▲- - -▲); and $dA(dT)_{10}$ (■——■) were also included. 1 pmol of $^3$H-TTP is equal to 2000 ct/min.

transferase, similar to that described by McCaffrey, Smoler and Baltimore (1973). The kinetics of the endogenous and template stimulated enzyme activities are also shown in Fig. 2. As can be seen, the high speed pellet contains an endogenous DNA polymerase reaction. A striking preference is also observed for an RNA-like template $rC(dG)_{12}$, which is considered to be specific for reverse transcriptase (McCaffrey *et al.*, 1973). Neither of the other synthetic templates $rA(dT)_{10}$ and $dA(dT)_{10}$ led to substantial stimulation of enzyme activity. These templates are less specific for reverse transcriptase activity than $rC(dG)_{12}$, and the extraction applied to the cells does not regularly yield high activities of DNA-dependent DNA polymerase (see for example, Fig. 3, lower panel).

*Reverse transcriptase activities in marrow cultures from patients in relapse and remission*

We have assessed RNA-dependent DNA polymerase activity (reverse transcriptase) in marrow cells from patients with leukaemia as a function of disease status. Marrow cells obtained during relapse or remission were assayed for enzyme activity. In addition, we asked whether or not the enzyme activity could be influenced by external factors; to this end we measured activity in cells after 7 days in culture. Reverse transcriptase was either absent or below the detection level in the marrow of 3 of the patients without malignant disease, either before or after culture. However, enzyme activity was found in every patient with leukaemia. A typical result is presented in Fig. 3, which depicts product analysis of enzyme activity from cells of a patient in relapse (top panel) and a patient in remission (bottom panel) before and after 7 days in culture. Enzyme activity was obtained before culture in the cells of both patients although the activity was lower in the patient in remission. In both instances the enzyme activity was increased strikingly after culture and in the case of the patient in remission reached levels comparable with those seen in relapse. The Table contains a summary of the data on the other patients. Reverse transcriptase activity is expressed as radioactivity summed over the regions of the caesium sulphate density gradient containing molecules with densities of RNA or RNA-DNA hybrids. Reverse transcriptase activity was found to be associated with marrow cells of all patients with leukaemia and in every instance increased following culture. However, both the highest initial levels and greatest increases were observed with marrow specimens from patients with ALL. Remission induction abolished neither the enzyme activity nor the capacity of cells to respond to culture conditions by increased enzyme levels.

FIG. 3.—Caesium sulphate density profiles of $^3$H-TTP labelled enzymatic products from marrow of a leukaemic patient in relapse (upper panel) and in remission (lower panel). Analysis of 5 × 10$^7$ cells before (●——●) and after (○——○) culture for 7 days. Activity in fractions 1–14 inclusive was considered to be characteristic of RNA and RNA-DNA hybrids; this was used as a measurement of reverse transcriptase activity (Table).

The profile in the upper panel was obtained from the marrow of a 19-year old male with ALL; the marrow was hypercellular with 89% lymphoblasts. Recovery after culture was 85% of nucleated cell input.

The profile of the lower panel was obtained using marrow from a 19-year old female with ALL in firm haematological remission for one year. Her marrow was normocellular with 44% granulopoietic precursors, 45% erythroblasts, 3% lymphocytes, 2% monocytes, 5% plasma cells and 1% unidentified blasts. Recovery of nucleated cells after culture was 85% of input.

TABLE.—*Endogenous Reverse Transcriptase Activity of Marrow Cells from Human Leukaemic Patients*

| Diagnosis | Clinical status | Number of samples tested | Reverse transcriptase activity (ct/min 5 × 10$^7$ cells)* Before culture Mean | Before culture Range | After culture Mean | After culture Range | Mean increase in activity after culture (Mean after culture / Mean before culture) |
|---|---|---|---|---|---|---|---|
| ALL | Relapse | 3 | 4600 | 4100–5700 | 35000 | 13000–55000 | 7·5 |
| | Remission | 4 | 1700 | 500–3800 | 23000 | 11000–45000 | 13·5 |
| AML | Relapse | 1 | 700 | 700 | 1600 | 1600 | 2·3 |
| | Remission | 1 | 700 | 700 | 2400 | 2400 | 3·4 |
| CML | Blast crisis | 1 | 5200 | 5200 | 14000 | 14000 | 2·7 |
| | Remission after† blast crisis | 1 | 2600 | 2600 | 8000 | 8000 | 3·1 |

* Total $^3$H-TTP incorporated into nucleic acid with densities characteristic of RNA and RNA-DNA hybrid (Fig. 1).

† Marrow status returned to that of CML.

### DISCUSSION

These studies yielded 2 new pieces of information about reverse transcriptase activity in human leukaemia. First, the activity increases when the cells are cultivated for 7 days. While the culture conditions were those devised for the growth of leukaemic cells (Aye *et al.*, 1973), the various components of the media have not been tested systematically as requirements for stimulation of reverse transcriptase. In some instances increased reverse transcriptase activity was observed in cultures containing haemachromatosis-LCM while in others the effect was seen in its absence. Second, enzyme activity was found in marrow cells obtained from patients in remission and increased following culture, often reaching the levels seen in patients in relapse. The differential counts on these remission marrows were close to those of normals; accordingly, our findings are consistent with the view that presence of the enzyme activity cannot be attributed to the presence of large numbers of undifferentiated cells (Bobrow *et al.*, 1972).

Reverse transcriptase activity is usually considered to be associated with leukoviruses. The enzyme activity observed in our experiments was stimulated strikingly by the artificial template $rC(dG)_{12}$, a finding consistent with the view that this activity may also be associated with leukoviruses. Particles found in leukaemic cells and the supernatants of suspension cultures of these cells will be described in a subsequent publication. These particles have biochemical and morphological characteristics similar to those of leukoviruses of murine or avian origin.

Supported by Grant MT-1420 from the Medical Research Council of Canada, Grant 236 from The Ontario Cancer Treatment and Research Foundation, and The National Cancer Institute of Canada.

### REFERENCES

AYE, M. T., TILL, J. E. & MCCULLOCH, E. A. (1972) Growth of Leukemic Cells in Culture. *Blood*, **40**, 806.

AYE, M. T., TILL, J. E. & MCCULLOCH, E. A. (1973) Studies of Leukemic Cell Populations in Culture. *Blood*. In press.

BAXT, W., HEHLMANN, R. & SPIEGELMAN, S. (1972) Human Leukaemic Cells Contain Reverse Transcriptase Activity Associated with a High Molecular Weight Virus-related RNA. *Nature, New Biol.*, **240**, 72.

BOBROW, S. N., SMITH, R. G., REITZ, M. S. & GALLO, R. C. (1972) Stimulated Normal Human Lymphocytes Contain a Ribonuclease-sensitive DNA Polymerase Distinct from Viral RNA-directed DNA Polymerase. *Proc. natn. Acad. Sci. U.S.A.*, **69**, 3228.

GALLO, R. C., YANG, S. S. & TING, R. C. (1970) RNA Dependent DNA Polymerase of Human Acute Leukaemic Cells. *Nature, Lond.*, **228**, 927.

HASSELBACK, R., CURTIS, J., SOOTS, M., ROBERTSON, G. L., COWAN, D. H. & HART, G. D. (1967) The Influence of Morphology on Prognosis in Acute Leukemia. *Can. med. Ass. J.*, **96**, 1610.

ISCOVE, N. N., SENN, J. S., TILL, J. E. & MCCULLOCH, E. A. (1971) Colony Formation by Normal and Leukemic Marrow Cells in Culture: Effect of Conditioned Medium from Human Leukocytes. *Blood*, **37**, 1.

MCCAFFREY, R., SMOLER, D. F. & BALTIMORE, D. (1973) Terminal Deoxynucleotide Transferase in a Case of Childhood Acute Lymphoblastic Leukemia. *Proc. natn. Acad. Sci. U.S.A.*, **70**, 521.

SARNGADHARAN, M. G., SARIN, R. S., REITZ, M. S. & GALLO, R. C. (1972) Reverse Transcriptase Activity of Human Acute Leukaemic Cells: Purification of the Enzyme, Response to AMV 70S RNA, and Characterization of the DNA Product. *Nature, New Biol.*, **240**, 67.

SPIEGELMAN, S., BURNY, A., DAS, M. R., KEYDAR, J., SCHOLM, J., TRAVNICEK, M. & WATSON, K. (1970) Characterization of the Products of RNA-directed DNA Polymerases in Oncogenic RNA Viruses. *Nature, Lond.*, **227**, 563.